# Acute skin toxicity and self-management ability among Chinese breast cancer radiotherapy patients: a qualitative study

**DOI:** 10.1007/s00520-024-08583-3

**Published:** 2024-05-30

**Authors:** Xiaomeng Lu, Yunteng Yin, Wenhui Geng, Lei Liu, Fengxia Liu, Zhenye Zhang

**Affiliations:** 1 Radiotherapy Department, The Fourth Hospital of Hebei Medical University, Shijiazhuang, China; 2 Department of Otolaryngology, The Fourth Hospital of Hebei Medical University, Shijiazhuang, China; 3 Department of Breast Centre, The Fourth Hospital of Hebei Medical University, Shijiazhuang, China; 4 Department of Nursing, The Fourth Hospital of Hebei Medical University, Shijiazhuang, China; 5 Department of Hospital Management, Shenzhou Hospital, Shenzhou, Hebei Province China

**Keywords:** Breast neoplasm, Radiotherapy, Radiodermatitis, Quality of life, Self-management, Qualitative research

## Abstract

**Objectives:**

Radiation dermatitis is the most common reaction to radiotherapy, almost all breast cancer patients receive radiotherapy on an outpatient basis. Currently, there are no studies on the experience of radiation dermatitis and the ability to self-manage it. Therefore, we aimed to use qualitative approaches to gain a deeper understanding of the actual experiences and self-management ability in order to provide a reference for further improving the effectiveness of self-management and to optimize symptom management strategies.

**Methods:**

A descriptive qualitative study was conducted using purposive sampling to select 17 breast cancer patients undergoing radiotherapy. Semi-structured interviews were conducted from September to November 2023. The Colaizzi seven-step analysis method was used to classify the data into summarized themes.

**Results:**

Four themes were identified from the interview responses: (1) multiple self-reported skin symptoms in breast cancer patients with radiation dermatitis; (2) the multidimensional impact on patient’s quality of life, especially pruritus, ulceration; (3) the ability to self-manage radiation dermatitis: strong mental toughness, positive response, and self-doubt; (4) challenges faced: concerns about radiotherapy side effects and recurrence, targeted symptom management and continuity of care after the radiotherapy.

**Conclusions:**

Healthcare professionals should consider patients’ self-reported symptoms when assessing radiation dermatitis. For pruritus and pain, we can enhance precision symptom management to improve patients’ quality of life. By utilizing information technology tools, we can increase breast cancer patients’ ability and confidence in managing radiation dermatitis effectively while enhancing accurate symptom management during radiotherapy.

## Introduction

Breast cancer is the most common cancer in women [1, 2]. Most breast cancer patients receive adjuvant radiotherapy (RT) after breast-conserving or mastectomy to reduce local recurrence rates and improve overall survival [3–5]. Radiation dermatitis (RD) is the most common complication of radiotherapy, 97.3% of patients experience radiation dermatitis during radiotherapy, and 62% of breast cancer patients experience grade 2 or higher radiation dermatitis [6]. These skin reactions, ranging from erythema to desquamation, in severe cases, and ulceration, are debilitating and often hurt patients’ quality of life. However, at present, the assessment of radiodermatitis is predominantly carried out by healthcare providers, and there is potential heterogeneity between assessors [7]. In clinical practice, clinicians tend to focus on treatment outcomes and only severe toxicity [8], clinicians significantly underestimate all skin symptom assessment items compared to patients [9, 10], and symptoms commonly experienced by patients during radiotherapy, such as hyperpigmentation, dry skin, and pain, are often overlooked [8]. Except for surgery, the majority of breast cancer treatments are delivered on an outpatient basis, highlighting the importance of patients’ self-management of radiodermatitis symptoms and the fact that most patients want to manage their own radiodermatitis [11]. Despite the high prevalence of radiodermatitis in this patient population, there is no standardized tool available to assess skin symptoms from the patient’s perspective. Most patient-reported outcome (PRO) tools for skin conditions are generic and require further validation before being used in breast cancer [12]. Multidimensional scales are needed to fully assess patients’ experience of radiodermatitis and their ability to self-manage it. Simply asking about physical symptoms like pain is not enough to capture all the changes in quality of life associated with skin changes. Currently, less is known about how patients perceive symptoms, cope with them, and what their dilemmas and needs are in terms of self-management. Therefore, this study was conducted using qualitative research methodology to better understand skin toxicity symptoms from the patient’s perspective. The aim was to explore various aspects of experiencing and managing radiation dermatitis associated with radiotherapy for breast cancer systematically and multidimensionally while identifying patients’ needs and abilities for self-management.

## Methods

### Participants, study design

Use of maximum difference purposive sampling to get the most information from the study subjects.

Subjects were selected from postoperative breast cancer patients in a tertiary hospital in Shijiazhuang City, taking into account gender, age, education level, type of surgery, and number of radiotherapy sessions. The inclusion and exclusion criteria are shown in Table 1. The sample size was based on the criterion of data saturation. When 15 interviewees were collected, no new information emerged, and to further confirm whether data saturation had been reached, two additional interviewees were conducted, and the interviewees were stopped when no new themes emerged. All patients interviewed signed an informed consent form. The study was approved by the Ethics Committee of the Fourth Hospital of Hebei Medical University in accordance with the declaration of Ministry of Health’s Measures for Ethical Review of Biomedical Research Involving Human Beings (2016), the WMA Declaration of Helsinki (2013), and the CIOMS International Ethical Guidelines for Biomedical Research on Human Subjects (2002) (Registration number: 2023KS117).

**Table 1:** Inclusion and exclusion criteria

Inclusion criteria	Exclusion criteria
(1) At least 18 years old	(1) Complicated with serious diseases of the heart, lung, kidney, liver, and other organs
(2) Diagnosed with breast cancer based on pathological examination	(2) Patients with serious mental or psychological diseases
(3) Undergoing radiotherapy or having completed radiotherapy in the last 30 days	(3) Language communication barriers
(4) Informed consent and voluntary participation in this study	(4) Skin allergy

### Determine the interview outline

Based on the results of the literature review, an interview outline was initially developed, and two outpatient breast cancer radiotherapy patients were selected for pre-interviews (pre-interview data were not included in the analysis of results). After listening to the views of the interviewees and taking guidance and suggestions from clinical nurse specialists, radiotherapists, and other professionals, the final formal interview outline was as follows: (1) Can you tell me how your skin changed during radiotherapy? (2) Can you describe your experience with radiation dermatitis? (3) How does radiation dermatitis affect your quality of life? (4) How do you manage your radiation dermatitis? (5) What are your needs for managing your radiation dermatitis? (6) And finally, is there anything else you would like to add?

### Data collection

#### Initial screening review of electronic medical records for eligible patients

Obtained general patient information includes age, marital status, education, occupation, skin color, stage, type of surgery, number of radiotherapy fractions, total radiation dose, number of radiotherapy treatments at the time of interview, and highest physician-rated RTOG grade. The researchers conducted semi-structured, in-depth face-to-face interviews with enrolled patients. The interviews were tape-recorded while the patient’s non-verbal behavior was observed and field notes were taken. The interviewees lasted approximately 20 – 40 min. The principle of the interview is that the patient explains his/her true feelings until no new information emerges.

### Data analysis

Two trained researchers collated the data within 24 h after the interview, recording non-textual data such as emotions, words, and pauses during transcription. In case of disagreement, they will be returned to the interviewee for confirmation. The researcher used Nvivo 12.0 software to save and analyze the transcribed text. Data were analyzed using Colaizzi’s seven-step method.

## Results

### Sample characteristics

A total of 17 female breast cancer patients, aged between 23 and 68, were interviewed in this study. The specific information is shown in Table 2.

**Table 2:** General information of respondents (*n* = 17)

NO	Age	Educational level	Marital status	Occupation	Skin color	Pathological stage	Type of surgery	Number of radiotherapy fractions	Total radiation dose	Number of radiotherapy fractions at the time of the interview	Highest physician-rated RTOG grade
P1	39	Regular college course	Currently married	Medical staff	Yellow skin	Stage IIIC	Breast reconstruction	25	50 Gy	20	2
P2	47	Postgraduate	Currently married	Teacher	Yellow skin	Stage II A	Modified radical mastectomy	25	50 Gy	21	2
P3	45	Regular college course	Currently married	Teacher	Yellow skin	Stage I	Breast-conserving surgery	25	60 Gy	20	2
P4	34	Junior college	Currently married	Freelance work	Yellow skin	Stage II B	Breast reconstruction	24	48 Gy	4	1
P5	50	Primary school	Currently married	Unemployed	Yellow skin	Stage III B	Modified radical mastectomy	25	50 Gy	25	1
P6	44	Regular college course	Currently married	Teacher	White skin	Stage II B	Modified radical mastectomy	25	60 Gy	24	2
P7	68	Senior high school	Currently married	Teacher	Yellow skin	Stage II B	Modified radical mastectomy	25	50 Gy	14 days after radiotherapy	2
P8	45	Regular college course	Currently married	Retired people	Yellow skin	Stage III B	Modified radical mastectomy	25	50 Gy	25	1
P9	42	Regular college course	Currently married	Unemployed	Yellow skin	Stage II B	Modified radical mastectomy	25	50 Gy	25	1
P10	23	Postgraduate	Never married	Student	Yellow skin	Stage I	Breast-conserving surgery	25	60 Gy	21	1
P11	44	Regular college course	Currently married	Unemployed	Yellow skin	Stage III B	Modified radical mastectomy	28	64.5 Gy	30	1
P12	55	Regular college course	Currently married	Teacher	Yellow skin	Stage II B	Modified radical mastectomy	25	50 Gy	20	1
P13	45	Primary school	Divorced	Staff	Yellow skin	Stage II A	Breast-conserving surgery	25	60 Gy	24	2
P14	54	Junior college	Currently married	Hairdresser	Yellow skin	Stage I	Breast-conserving surgery	25	50 Gy	24	2
P15	68	Primary school	Currently married	Retired people	Yellow skin	Stage I	Breast-conserving surgery	15	49.5 Gy	15	1
P16	67	Primary school	Currently married	Farmer	Yellow skin	Stage II A	Modified radical mastectomy	25	50 Gy	12 days after radiotherapy	2
P17	33	Regular college course	Currently married	Unemployed	Yellow skin	Stage II A	Breast reconstruction	25	50 Gy	25	1

### Interview results

**Fig. 1:**
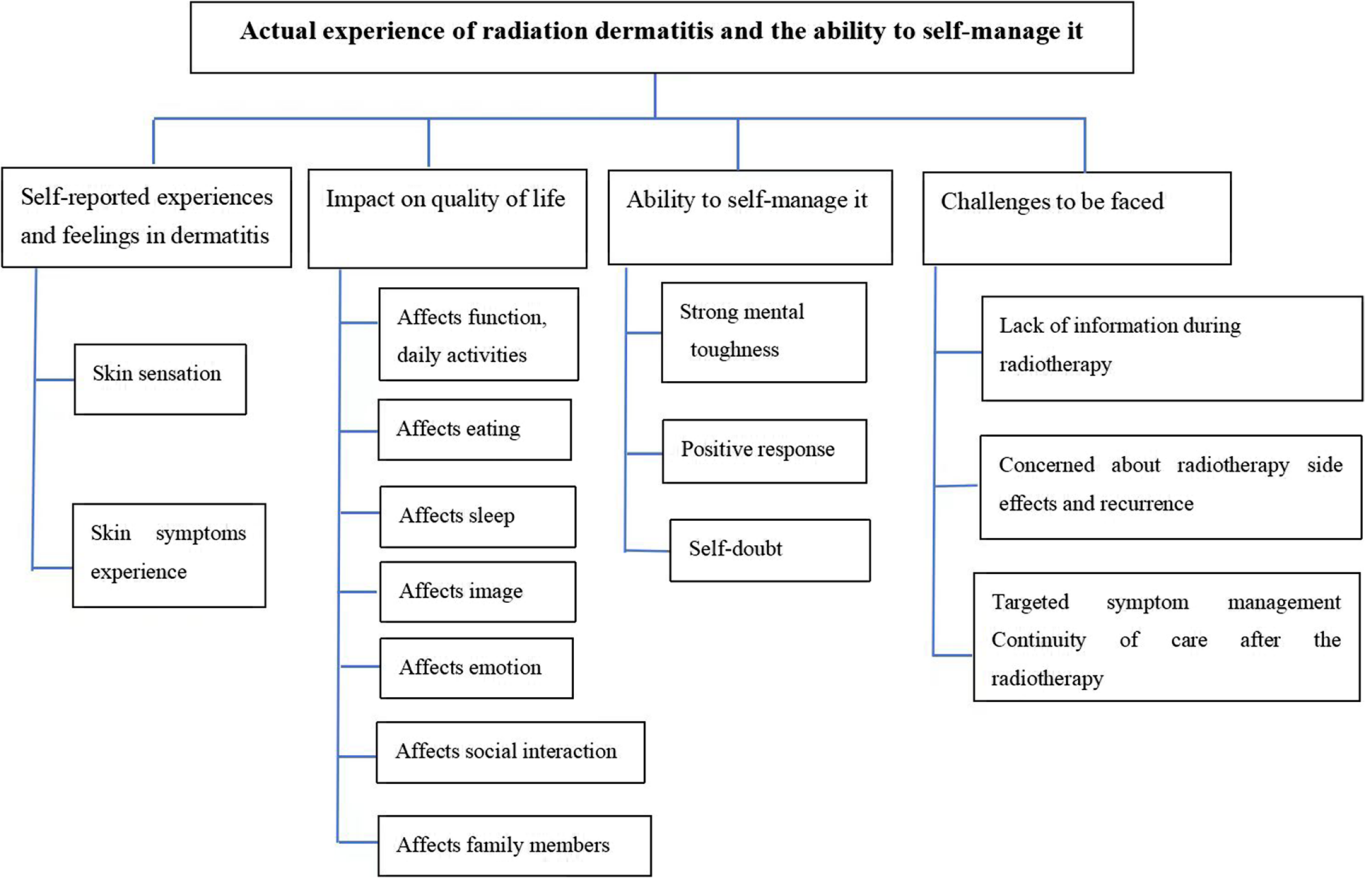
Themes and sub-themes of breast cancer patients acute skin toxicity and self-management ability

Four themes were formed: “self-reported experiences and feelings of radiation dermatitis”; “impacts on quality of life”; “ability to self-management it”; and “challenges to be faced.” Themes and sub-themes are shown in Fig. 1.

### Theme 1: Self-reported experiences and feelings of radiation dermatitis

#### Skin sensation

##### Hyperpigmentation and erythema of the skin

The most common skin color changes mentioned were hyperpigmentation and erythema. Of the interviewees, 94.2% mentioned skin hyperpigmentation in the radiation field, while 76.5% suffered from skin reddening. P1: “The skin showed a little bit of rpigmentation on the 15th day, in the armpits and neck. At the 18th day I felt a little bit of redness in the chest.” P12: “At the 7th day there weren’t any differences, but the color got darker and darker. Sometimes, when it was hot it got red.” P10: “The skin was a little red at the beginning and then I noticed that it turned a little bit hyperpigmented.”

##### Burning sensation of the skin

P2: “The skin exposed to radiation has been higher in temperature than the normal skin since the third week. Additionally, it felt hotter gradually.” P16: “It felt terribly burning when touching the armpit or its front.”

##### Swelling sensation of the skin

P11: “I felt the armpit and the chest swollen. I can feel it was swelling.” P12: “The armpit and the chest felt like swollen.” P3: “It was about in the 7th radiotherapy when the breast was so swollen and distended that the affected breast was bigger than the healthy …” P15: “It was just a little bit bigger. It always felt like crashing, swollen and hard…”.

##### Tightness of the skin

P7: “The skin was tight as if the flesh was being pulled together… I hunched over but dare to straighten my back.” P8: “In the last round of radiotherapy, I feel that the skin was tight, especially at the surgical incision. It was a bit stretched out when I lift my arm.” P10: “About 8 times, the skin was accompanied by this kind of tightness. It was a bit stretched when I lift my arm.” P9: “It felt tight and even worse than it did after operation…Maybe it had something to do with less rehabilitation exercise during radiotherapy.”

##### Dryness of the skin

P9: “I felt that the skin is too dry. When it’s dry I felt very hard, it always felt like I’m losing a layer of dry skin.”

##### Skin ulceration

Of the 17 interviewees, the ulcers were all in patients with total mastectomy (Four cases). P2: “After19 times, the ulcers outbroke in armpit, and then oozed.” P7: “Since the 22th radiotherapy, there had been more blisters in the armpit, and the biggest was as big as a walnut…” P8: “One or two days after the end of treatment, there was a small ulcer in the armpit.” P16: “1 week after the final radiotherapy, the armpit was a bit itchy, I didn't dare to scratch it, and then I took alcohol wipes and dipped it, then it broke the skin, there were black scabs, the chest also got two blisters, oops, a rub will break.”

##### Poor skin sensitivity

P11: “I can’t felt my skin…As the therapy went on, the feeling get even worse.” P7: “When I touched my skin, it felt like kind of numb. All of this began after surgery, but got even much worse after the radiotherapy.” P16: “My skin couldn’t feel cool when I apply a cold water bag on it.”

#### Skin symptom experiences

##### Skin itching

Patients report symptoms varied in both location and degree of itching. P1 (breast reconstruction): “I felt a little itchy during the 18th radiotherapy, but it was not as severe as to affect my sleep. After 19th it was noticeably extra, while it continued to be particularly on the chest until the 24th time.” P6 (modified radical mastectomy): “My neck was more itchy, but it was mild and didn’t affect sleep.” P9 (modified radical mastectomy): “The skin of the chest was not very itchy, but in hot days, it felt especially itchy. After about 17 times, the skin itches unbearably, and I had to get up to deal with it in the midnight.” P10 (breast-conserving surgery): “My skin felt tight and itchy, when I underwent the 15th to 21st radiotherapy. My skin kept itching all the time…” P13 (breast-conserving surgery): “The skin of my breast was slightly itchy, but I could stand it…”.

##### Pain caused by skin ulcers

The pain is mild in non-ulcerated patients and mainly tingling. It is severe after rupture. P4 (breast reconstruction): “There was a tingling sensation in the breast during radiotherapy.” P3 (breast-conserving surgery): “I felt a little painful inside the breast. It felt like pins and needles, but only lasted about a second and then it didn't hurt anymore.” P15 (breast-conserving surgery): “When I felt the breast swollen, I touch it and it hurts, but not very much.” P2 (modified radical mastectomy): “Since the 19th radiotherapy, the ulcer in my armpit broke and it started to hurt.” P7 (modified radical mastectomy): “On the 22th day of radiotherapy, I had a lot of blisters on my armpit. I felt so painful that I couldn't sleep at night.” P16 (modified radical mastectomy): “At the beginning, I felt a little bit of painful, and it didn’t matter. As the radiotherapy progressed, I felt more and more painful in the chest burst, so that I could not turn around.”

##### Friction in the armpits

P1: “When I started radiotherapy, my armpit had a feeling of friction. I felt particularly painful when the skin rubbing back and forth. My armpit felt very tight. If my arm dropped down, it would feel like something was stuffed inside.”

### Theme 2: Impacts on quality of life

#### Affects function, daily activities

##### Effects on household activities

P2: “I couldn’t do anything mainly because mostly due to my skin ulcers. I couldn’t cook for my family, and I couldn’t complete my house chores, either.” P6: “Especially when I went to the kitchen to cook, the high temperature from stove caused pain in my skin…and I could not bear it.” P1: “My activity is also a bit limited…holding a mobile phone for a long time, or doing some housework is not very convenient.”

##### Effects on body movement

P1: “Before the 15th time, I could turn to each side, but after 15 times there was a feeling of pressure when I turned to the affected side. It was like some endema, and I felt not very comfortable.” P15: “The skin was always painful, and I couldn't exert any pressure even when I turn…”.

##### Effects on upper limb function

P8: “In the later stage, I felt the skin was tight, especially at the surgical incision. It was a bit stretched when I lifted my arm. All the skin in the armpit was a bit tight, I used to be able to lift it in one go, but now I can only lift it after a few attempts.” P13: “I felt it was fine when I lifted my arm before radiotherapy, but I felt a bit restricted in lifting it after radiotherapy, not as smoothly as before.”

#### Affects eating

P2: “I’m afraid to eat heavy flavors or spicy food, because I may sweat after eating it. I can’t eat it no matter how much I want.” P11: “Oops, since those two days (26–28 times), I’ve been in a nasty mood and I have no appetite either!” P16: “Is it because I ate some meat that my skin got serious? It felt like I had eaten something hairy and all of a sudden my skin broke out and got worse and worse.”

#### Affects sleep

Interviewees reported that pain and itching had a greater impact on sleep. P2: “My armpit skin ulcers were particularly painful and affected my sleep.” P11: “Oops, after 26–28 times the itching got so bad, I was annoyed and couldn't sleep well just because of it, it was hard and unspeakable.” P12: “I have been treated for 20 times, while I only feel itchy skin, and I had trouble sleeping last night.”

#### Affects image

P1: “When I was outside today, the column was brighter like a mirror, when I looked at my neck after zipping up my shirt, it was extra red. It was embarrassing.”

#### Affects emotion

Itching and ulcers have a greater effect on emotion. P2: “It’s the ulcer that affects my mood. I don’t want to do anything.” P2: “The ulcer in the armpit is oozing. For example, when the halter dress is bigger, it often gets particularly dirty.” P1: “It was unbearably itchy and I felt particularly annoyed yesterday.” P11: “After the 26th radiotherapy, the itching was so bad that I started to feel anxious, and I was so annoyed. I couldn't use my mobile phone and sleep well.” P7: “The ulcer in my armpit is oozing fluid, and it’s affecting my mood. I didn’t thought the radiation was so strong, and I feel particularly worried and psychologically anxious.”

#### Affects social interaction

Interviewees reported more social withdrawal. P7: “I seldom went out in half a month after the radiotherapy (because of the skin ulcers). My partner and I thought we would reject the visit of our relatives and friends.” P11: “I wish when I got better I would wear high-necked clothes. The collar could hide it. But I don’t go out now, because I'm afraid it will be damaged by the light and wearing clothes will rub my skin.” P14: “I haven’t told anyone about my illness. If I go out I don’t want them to see my skin, that's what I feel now.”

#### Affects family members

Interviewees expressed concern and guilt about their families. P2: “I don’t want to do anything when my skin ulcerated. I can’t rely on the elderly to cook for the family, while there is a sense of guilt. The skin always hurts, the more irritable I am, the more guilty to the family.” P11: “At first I was worried that my child might not accept, but now I have to keep my skin dry. I can feel that the impact on the child is quite big, and his spirit is hurt.”

### Theme 3: Ability to self-manage radiation dermatitis

#### Strong mental toughness

P2: “Overall, for me, it’s just a superficial injury. I don’t think it’s anything special. It’s not as serious as an internal injury, although it will affect some of my work…and my life, it’ll be fine after a few weeks, and I can hold on to it.” P11: “Relative to the whole condition, I can accept that it’s just a localized one. I feel that it will be fine after a long period. I believe that I can be as healthy as before after some time, half a year or more. Maybe the color will be a little darker, definitely a little different from this side, but it doesn't expand to the normal skin, that's all acceptable.” P12: “It’s a small thing compared to what I’ve experienced before.” P7: “The skin is just red, and it’s acceptable. That it will slowly recover. It’s okay to have scars on the breast and it won’t be visible. It’s okay if it slowly recovers to normal.”

#### Positive response

Interviewees demonstrate strong self-management of symptoms, including keeping the skin clean, observing the skin daily, and planning the use of skin protection products. P1: “I apply the cream depending on the time of day. 2 or 3 times a day, thicker layer at night before going to bed. I don’t apply it 4 h before radiotherapy. If I have radiotherapy at noon I would apply a thinner one at 7 or 8 in the morning.” P8: “Prior to spray, I check my skin carefully every day to see if there are any problems. The neck might be a bit red and a bit itchy, and I dare not scratch it. So I just rinse it with water to cool it or turn on the air conditioning to blow on it, and then it’s fine.”

#### Self-doubt

Some interviewees have high expectations and are not satisfied with the behavior they are paying for. P10: “I will give myself 8 out of 10 points for protecting my skin. I just do my best to protect it, but it seems like that I’m not protecting it well, because other people who are having radiotherapy at the same time, they didn’t have skin problems.” P14: “I’m now almost out of aloe vera, and my skin might break out. I’m kind of regretting it now… If I use some aqueous cream or something else, my skin might be better. I’m a bit regretful that I didn’t buy the other ones.”

### Theme 4: Challenges to be faced

#### Lack of information during radiotherapy

Respondents expressed a lack of knowledge about radiotherapy and some information about precautions to be taken. P4: “Every time you have radiotherapy, the radiotherapy waxy film (tissue-compensating film) that protects the skin or…” P6: “I don’t know why my skin is getting darker and red.” P13: “Can I exercise while I’m having radiotherapy? If I do, will it cause edema in my upper limbs?”.

#### Interviewees were most concerned about radiotherapy side effects and recurrence, and they were more concerned at the start of radiotherapy

##### Worries about skin problems

P2: “My main concern is that the skin does not get normal again. Is there anything I can do to make it heal slowly because I'm afraid it will get worse.” P11: “I don’t take exercise or move around all the time because they might stretching the skin under my armpits.” P12: “I’m worried about whether my skin will go normal in the future.”

##### Worries about edema

P12: “I can feel a little bit of swelling in the neck, but it’s not very much, and I'm just worried that this cervical lymph won't return and it's hard to go down.”

##### Worries about implant contracture

P1: “When I touch the implant inside the breast, it feels a bit hard. I think there’s a feeling of contracture. I’m worried that it would be more serious in the later stage. Whether implants in the breast can deform, whether it will affect the appearance. I was so afraid to take it out…” P4: “I am afraid that the prosthesis will become inflamed or contracted, it will be a problem if I have to take it out again…”.

##### Fear of recurrence

P13: “Is it a recurrence? Sometimes I will think…I felt especially scared.” P15: “I thought the redness and swelling of the breast indicated the metastasis and recurrence…honestly, it feels like it’s progressing.” P11: “The radiotherapy will be over soon, but I'm still worried about a recurrence. If I get a relapse, I will have to go through all the same things I have gone through, and get a double blow both physically and psychologically.”

##### Worries at the start of radiotherapy

P8: “I was particularly worried at the start…I watched every day, meanwhile my husband was also particularly worried if there was a small problem, something like a red spot…, I’d hurry to see if it was all right.” P12: “Worried at the start of radiotherapy, and I was anxious when I heard my fellow patients get some skin problems.”

#### Targeted symptom management and continuity of care after the radiotherapy

##### Targeted symptom management

P9: “Itchy skin is more unbearable, I hope the healthcare can help to relieve it.” P11: “After 26–28 radiotherapy treatments, my skin was so itchy that I got irritable.” P7: “After 22 radiotherapy treatments, more blisters appeared under the armpits. After finishing the radiotherapy treatment, the skin hurted so much at night that I can’t sleep. It hurts all the time, my activities are limited, and I can’t sleep. I feel anxious.”

##### Continuity of care after the radiotherapy

P4: “If there is a problem after radiotherapy, I hope I can contact the healthcare provider to see how to deal with it. I can’t deal with it myself.” P14: “I’m sure I’ll need to take a skin-breaking ointment with me when I'm finished. If it doesn’t look good I'll call or come back.” P14: “What should I do if I've finished my radiotherapy, if I’ve got a skin ulcer after I go home.” P3: “I don’t know what my breasts will look like at the end of radiotherapy and how long it will take to recover well.”

## Discussion

### Emphasize patients’ self-reported experience of radiodermatitis symptoms and construct self-reported outcome indicators for radiodermatitis in breast *cancer* patients

In clinical practice, healthcare providers assess the severity of RD every week in the outpatient setting using the Common Terminology Criteria for Adverse Events (CTCAE version 4.0 or 5.0) or the Radiation Therapy Oncology Group (RTOG). The CTCAE and RTOG only assess the severity of erythema, desquamation, or ulceration [13], and neither scale incorporates patients’ self-reported symptoms. There are significant differences between clinician-reported outcomes (CROs) and patient-reported outcomes (PROs) when assessing RD, CROs alone are insufficient to capture the impact on patients’ quality of life [14]. The results of previous studies highlight the need for improved assessment of RD and support to develop a new tool with patient and clinician components [9, 15]. Previous assessment tools such as the Dermatology Quality of Life Index and Skindex-16 have been used to capture PROs with RD, but a breast-specific tool is better for capturing the symptoms experienced by patients undergoing radiotherapy [16]. Although the validated RISRAS scale includes both clinician and patient assessments, it is not specific for breast cancer radiodermatitis too [17]. In recent years, patient-reported outcomes have been increasingly recognized as a way to improve practice through better communication and improved symptom management and identification of patient care needs [18–20]. It is defined as any state of a patient’s condition that comes directly from the patient, without interpretation by the clinician or anyone else [19]. Studies have confirmed that patient reports of their condition are more direct than physician reports and can identify abnormal symptoms early in treatment [8, 21]. An increasing number of studies are incorporating patient self-reported symptoms with health-related quality of life as an indicator of outcome [22, 23].

The use of patient-reported outcomes (PROs) in clinical practice enhances the timely recognition of symptoms, facilitates patient engagement in clinical decision-making, and improves the overall healthcare experience [24–26]. Incorporating a self-reported tool for outpatient radiotherapy patients could offer targeted education and resources to enhance patient self-management and enable real-time collection of patient-reported outcomes for early detection of potentially serious adverse events [27]. Therefore, this study aims to investigate the symptoms associated with radiation dermatitis and its impact on the quality of life in breast cancer patients, aiming to inform the development of a self-reported outcomes tool specifically designed for radiation dermatitis.

In this study, hyperpigmentation and erythema were identified as the most frequently reported symptoms, which aligns with previous research findings [9, 11, 28]. Additionally, patients with radiation dermatitis experienced various skin sensations such as swelling, burning sensation, tightness, dryness, and eruptions. Symptomatic experiences including itching, pain, and axillary friction were also present. Radiotherapy causes early capillary dilation, resulting in increased vascular permeability, skin congestion, and mild erythema, if damage extends into the dermis, the erythema worsens. Simultaneously, the body protects basal cells from further damage by releasing large amounts of melanin into the bloodstream, resulting in local hyperpigmentation. As injury progresses, damage occurs to accessory organs like sebaceous glands, sweat glands, and hair follicles leading to skin dryness, tickling,itching, and burning. Inflammation spreads to peripheral blood vessels causing edema [29]. In this study, the presence of swelling was reported among both breast-conserving surgery and modified radical mastectomy patients. A multicenter cohort study found that 451 (26.2%) out of 1723 patients reported acute breast edema [30], but patients’ self-reported breast edema was less severe than physicians’ (*p* < 0.001) [30, 31]. A pooled analysis of three trials showed that pain was common in radiotherapy patients, with reported rates ranging from 23.3 to 83.6% [14, 30, 31], and it was more commonly reported by patients themselves. Patients also experienced some degree of pruritus, which has been associated with serine proteases (e.g., KLK, matriptase), prostaglandins, or trypsin-like enzymes in radiation dermatitis. Genotyping of single nucleotide polymorphisms (GSTA1 rs3957356-CT, MAT1A rs2282367-GG) may be linked to the degree of pruritus in radiation dermatitis [32]. In one study, the incidence of patient self-reported pruritus was 83.4% [23], second only to erythema and pain. Severe pruritus significantly affected the lives and sleep quality of 106 patients (13.6%) [9]. Therefore, understanding the differences between patient-reported radiodermatitis and clinical practice is important for promoting changes in clinical practice and maximizing patients’ ability to self-manage their symptoms accurately while improving their quality of life.

### Maximize patients’ ability to self-manage, to strengthen accurate symptom management, and to improve patient’s quality of life

This study provides a better understanding of the experience during treatment for radiation dermatitis and its impact on quality of life from the perspective of breast cancer patients themselves. The results suggest that radiation dermatitis affects multiple aspects of quality-of-life among these patients as found in Schnur’s study [33]. Previous studies have also shown an impact on overall QoL due to “symptoms and sensations” related to skin conditions [34]. In-depth interviews conducted in this study revealed that pain caused by itching and wet scaling had a greater influence on QoL as it led to emotional distress and impaired daily functioning among patients. These findings support previous studies advocating for targeted symptom intervention programs aimed at improving QoL among affected individuals [21, 35]. The respondents in this study reported impaired daily functioning mainly due to skin tightness, which caused difficulty in lifting the upper limbs. Older female patients reported valuing function and health more than appearance, a finding similar to other studies in chronically ill or physically disabled populations [36, 37]. A survey showed that 53.2% of patients had a high need for radiation dermatitis management. Patients with symptoms of dryness, burning, irritation, roughness, and hyperpigmentation were 11.73, 7.02, 5.10, 4.27, and 2.80 times more likely to require treatment than patients without these symptoms [11]. Further research is needed to explore patients’ self-management skills and confidence in managing radiation dermatitis, this gap highlights the importance of identifying patients’ self-management skills particularly their confidence level. Patients’ creativity and problem-solving skills should be harnessed to improve their treatment experience. This study found that breast cancer outpatient radiotherapy patients had strong self-symptom management skills, they were able to manage mild symptoms on their own but needed help from healthcare professionals when symptoms were severe. Patients were more psychologically resilient and able to cope positively with radiation dermatitis. However, some participants reported concerns and barriers such as fear of starting radiotherapy or lack of confidence in controlling its side effects or fear of relapse. Their fear of radiotherapy was much worse than their actual experience with the treatment itself and its side effects, nevertheless, they often sought information to help control this fear [38, 39]. Therefore, it is important to fully exploit the intrinsic potential of breast cancer patients undergoing radiotherapy by providing information tailored according to their level of health literacy while making use of their existing skills and strengths.

### Using digital health technologies to improve future strategies for managing radiation dermatitis in breast *cancer* patients

In recent years, digital health technologies, such as global big data, telemedicine, wearable smart devices, and artificial intelligence, have gained widespread attention. Digital health-based Internet + , Internet of Things, and smartphone apps have been successfully developed to meet the needs of individual and population health levels, and improve the service quality and capacity of healthcare delivery systems [40]. They also play a supportive role in symptom monitoring and management for radiotherapy patients [41], with considerable potential for development as most patients receive radiotherapy on an outpatient basis. A report from Sweden demonstrated that the use of a digital information tool in VR technology for outpatient breast cancer radiotherapy patients reduced pain symptoms before, during, and 6 months after treatment while improving self-perception and health literacy. The information tool consists of two separate but related mobile devices: a VR app that provides guided tours, audio-visuals, and images to create the impression of being in the radiotherapy department before treatment and a pre-treatment information app that has been shown to increase patient understanding of their treatment, reduce anxiety, and improve patient follow-up rates [42]. In terms of symptom monitoring, a study in patients with prostate cancer showed early monitoring, reporting, and management of symptoms during radiotherapy using an interactive smartphone application (Interaktor) on a smartphone or tablet, with the intervention group reporting symptoms daily during and for 3 weeks after treatment via the app, which generated a text message to inform the doctor, who then monitored the patient’s condition. Monitoring of the patient’s condition showed that the patient’s burden in terms of emotional functioning, insomnia, and urinary symptoms was significantly reduced at the end of treatment and 3 months later, and that Interaktor could be an effective mHealth tool to facilitate the need for supportive care during treatment in cancer patients [43]. Moller et al. [44] validated the potential of longitudinal monitoring of patients’ symptoms during radiotherapy, where patients used their own electronic devices at home to self-report (ePRO) weekly changes in symptoms and quality of life during and after radiotherapy at week 4 and weeks 8, 12, and 24, confirming feasibility, usability, and acceptability. Therefore, the Internet and digital information technology have greatly increased the availability of information, and the use of digital health mobile apps to manage patients’ radiodermatitis symptoms is feasible. The design of a mobile app that allows patients to proactively report their radiodermatitis-related symptoms on their smartphones or tablets during or immediately after routine radiotherapy is expected to be a useful tool for patients to monitor and self-manage their symptoms and meet supportive care needs.

### Limitations

(1) The interviews were geographically limited; only patients attending a tertiary hospital in Hebei Province were interviewed, and although the study tried to cover as diverse a sample as possible, the study sample was still significantly biased. In addition, our interviewees were all Chinese, and that certain cultural backgrounds might influence perception of symptoms. In other populations with different backgrounds, the focus might be somewhere else. (2) The study was limited in time, and only one interview was conducted with each participant, which did not allow us to infer trends in radiation dermatitis over time and patients’ coping strategies. In the future, more regions and more longitudinal studies can be conducted to further broaden and deepen the understanding of the real experience of radiation dermatitis and the ability to cope with self-management.

## Conclusion

In this study, in-depth interviews with patients undergoing radiotherapy for breast cancer revealed multiple self-reported symptoms of radiation dermatitis in breast cancer patients and given the lack of standardized assessment methods for RD. It is important to include patients’ perspectives when assessing symptoms in future acute RD trials. Radiation dermatitis affects several aspects of quality of life, particularly itching and pain. Those with milder aspects of self-symptom management ability seek multiple symptom management strategies, and those with severe symptoms need help from healthcare professionals. In the future, it is expected that digital health technologies will be used to manage radiation dermatitis in breast cancer patients, providing a wide range of information support, as well as serving as a useful tool for patients to monitor and self-manage their symptoms and meet their supportive care needs.

## Data Availability

The datasets generated and/or analyzed in this study are available from the corresponding author on request. Reasonable requests for availability.

## Code availability

N/A.
